# Supplementary material for: Sanguisorba minor Scop.: An Overview of Its Phytochemistry and Biological Effects
Source: Plants (Basel). 2023 May 27;12(11):2128. doi: 10.3390/plants12112128 (PMC10255321; doi:10.3390/plants12112128)
Supplement: Supplementary file 1 [file plants-12-02128-s001.zip › plants-2335593-supplementary.pdf]

Materials and methods

Plant material

S. minor Scop. was harvested in May 2022 in the Cluj country village of Bucea, Romania which is located between 46°96′30″ N and 22°68′05″ E. The leaves, stems, and roots of the plant were collected separately and transferred to containers where 20 ml ethyl alcohol was added. The identification of *S. minor* Scop. plants were made at the Department of Pharmaceutical Botany at the University of Oradea, Faculty of Medicine and Pharmacy. A specimen was kept in the Herbarium of the Faculty of Medicine and Pharmacy Oradea, Romania, registered in NYBG Steere Herbarium, under the code: Uop 05 367- *S. minor* Scop.

Scanning Electron Microscopy (SEM) Analysis

The samples of *Sanguisorba minor* Scop. (roots, stems and leaves) were transported in sealed containers with ethyl alcohol, then transferred to Eppendorf tubes of 2 ml on 0.15 M phosphate buffer (PBS), for 1 hour, after that 2 washes were made with PBS 0.15M for 1 hour for each sample. After that, the samples were fixed with Hexamethyldisilazane reagent grade 99% from Sigma-Aldrich, for about 2 hours.

Dehydrating materials through fixation with Hexamethyldisilazane is also beneficial and essential for scanning analyses.

After that, they were mounted on brass supports (staves) with the help of self-adhesive carbon discs that allowed fixation and current conductivity. As a result, the Agar Sputter-Coater metallizer, the metallization of the samples was carried out, consisting in depositing a 10 nm layer of platinum on the surface of the samples, a process that helps in better reflection of electrons, resulting in a better image quality.

After metallization, the samples were examined using an image-capturing Hitachi SU8230 (Hitachi, Tokyo, Japan) scanning electron microscope operating at 30 kV. Images were taken of the samples allowed for the identification of the stomata, leaf surfaces, and roots.

Table S1. Biological activities of *Sanguisorba minor*

Health effects	Bioactive compounds	Sample type	Type of experiment	Main Outcomes	References
Anti-ulcerogenic	Saponins, tannins, flavonoids	Aqueous extract of aerial parts *S. minor*	*In vivo* induced gastric ulcer model in rats (Sprague-Dawley)	- gastric protection against the ethanol-induced gastric ulcer model in rats	[68]
Nutritional	Fatty acids (α-linolenic acid, palmitic, linoleic acid, tricosylic acids)	mixture of chloroform, methanol, and water extract of aerial parts of *S. minor*	gas–liquid chromatography	-high content of total fatty acids concentrations (22.2 g kg^{-1} DM) and α-tocopherol concentrations (85 mg kg^{-1} DM).	[86]

Nutritional and antioxidant activity	Organic acids Phenolic compounds	Aqueous extract of leaves from *S. minor*	*In vitro* test H-TAA, L-TAA, Hydroxyl radical (OH) and peroxyl radical (H_2O_2) scavenging	- the high value of H-TAA 368.91 ± 47.96 mg of Trolox equivalent 100 g^{-1} FW, -high total phenol levels (530.51 ± 21.12 mg 100 g^{-1} FW), -higher protector effect of DR against oxidative attack, with inhibition percentages of 57.75 ± 9.3% and best capacity for scavenging H_2O_2 64.35 ± 5.52%.	[56]
Neuroprotective	Flavonoids	Hydroethanolic extract aerial parts of *S. minor*	*In vitro* Male Wistar rats-treatment with scopolamine	- antiacetylcholinesterase effect -antioxidant activities in brain tissue,	[70]
Anticancer	Flavonoids	Ethanol extract of whole plant	*In vitro* scratch motility assay	- limits plasmin-mediated tumor cell motility *in vitro*, mostly due to quercetin-3-glucuronide.	[49]
Anticancer	Flavonoids	Maceration of aerial parts and roots of *S. minor* Methanol/water (80 : 20, v/v)	*In vivo* four human cancer cell lines: HeLa, HepG2, MCF-7 and NCI-H460, and PLP2.	- high inhibition of cell growth: HeLa (GI_{50} = 60–75 μg mL^{-1}), MCF-7 (GI_{50} = 81–199 μg mL^{-1}) and NCI-H460 cell lines (GI_{50} = 130–199 μg mL^{-1}).	[28]
Antioxidant and antimicrobial	Flavonoids Tannins	Leaves and roots of *S. minor* methanol/ water (80:20, v/v)	*In vitro Staphylococcus aureus, Bacillus cereus, Listeria monocytogenes, Pseudomonas aeruginosa, Escherichia coli , Enterobacter cloacae, Salmonella typhimurium, Aspergillus fumigatus, Aspergillus niger, Aspergillus ochraceus, Penicillium ochrochloron, Penicillium funiculosum, and Penicillium verrucosum var. cyclopium*	-the roots extract revealed a higher antibacterial capacity in comparison with leaves, especially against *S. aureus*, *B. cereus*, *L. monocytogenes*, and *S. typhimurium* -the roots extract contained a higher amounts of total phenolic acids (27.7 mg/g), total hydrolysable tannins (127.53 mg/g), total flavonoids (64.6 mg/g) and total phenolic compounds (219.81 mg/g).	[55]
Antioxidant	Phenolic compounds	Ethanol extract of roots, leaves and stems of *S. minor*	*In vitro* DPPH, FRAP, Folin Ciocalteu	-ethanolic roots extract exhibited high levels of DPPH radical neutralization (92.93% of inhibition), respectively	[4]

				3.89 mg GAE/mL by the Folin Ciocalteu and 10.81 μmol TE/g by FRAP method.	
Antimicrobial	Tannins Flavonoids Phenolic acids	Ethanol extract of roots, leaves and flowers of *S. minor*	*In vitro Staphylococcus aureus, Escherichia coli, Pseudomonas aeruginosa*	-the ethanol extract of *S. minor* Scop. leaves exhibited better antibacterial activity against all of the bacteria tested, especially on *Staphylococcus aureus*, with an inhibition zone of 15.33 ± 0.83 mm.	[44]
	Phenolic compounds	Maceration of aerial parts of *S. minor* methanol/water (80 : 20, v/v)	*In vitro B. cereus, L. monocytogenes, E. coli, S. typhimurium, En. cloacae, S. aureus.*	-*S. minor* Scop. roots extracts exhibited the ability to inhibit the growth of *B. cereus, En. cloacae, L. monocytogenes, S. aureus,* and *S. typhimurium*, at concentrations of 0.075 mg mL^{-1}.	[28]
Neuroprotective and antioxidant	Phenolic compounds	Three extracts: ethanolic extract, water extract and essential oil of aerial parts of *S. minor*	DPPH, enzymatic activity	-high inhibition value of AChE in the ethanolic (78%) at 1mg/ml.<br>- all three extracts showed antioxidant activity, but ethanolic extract and decoction have the highest value (93%).	[65]

DM- Dry matter, H-TAA- hydrophilic antioxidant activities, L-TAA- lipophilic antioxidant activity, FW- fresh weight, DR-deoxyribose, LC-MS- reversed-phase coupled to high-resolution mass spectrometry, HeLa- cervical carcinoma, HepG2- hepatocellular carcinoma, MCF-7- breast adenocarcinoma, NCI-H460- non-small cell lung cancer, PLP2- porcine liver cell primary culture, GI_{50}- extract concentration that inhibited 50% net cell growth, GAE-gallic acid equivalent, TE-Trolox Equivalent, AChE –acetylcholinesterase.
